# Supplementary material for: Predicting gene expression in the human malaria parasite Plasmodium falciparum using histone modification, nucleosome positioning, and 3D localization features
Source: PLoS Comput Biol. 2019 Sep 11;15(9):e1007329. doi: 10.1371/journal.pcbi.1007329 (PMC6756558; doi:10.1371/journal.pcbi.1007329)
Supplement: S1 Fig — Comparison of test AUCs for motif-only models using 500 bp, 1kb, or 2kb promoter windows. (PDF) [file pcbi.1007329.s001.pdf]

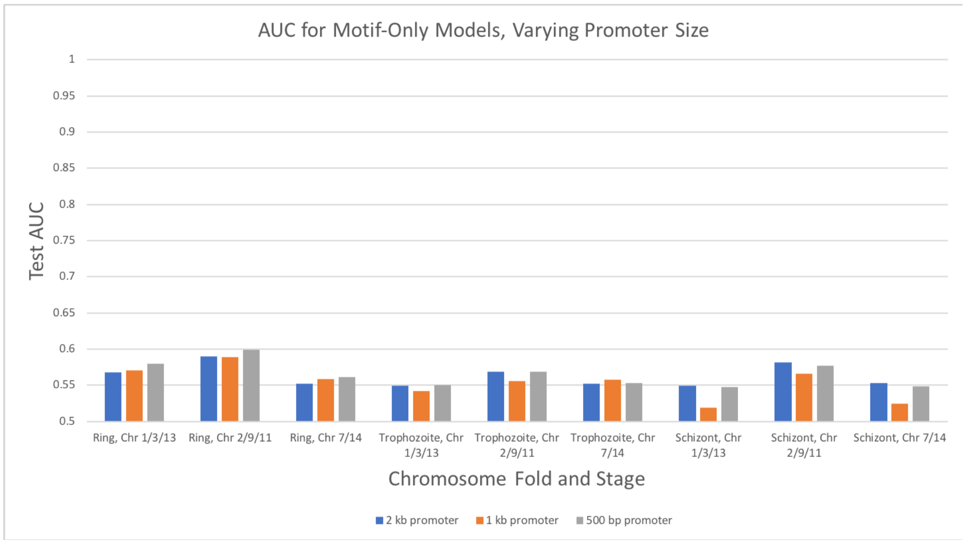

S1 Fig: Varying promoter size. The test AUC values obtained by training logistic regression models using motif-only features. Test errors are labeled by the data fold that was held out as test data. All models selected hyperparameters by cross-validation within the two folds of training data, using the best-performing hyperparameters to train a final model for evaluation on the test set. Motif features were determined using promoter windows of 500 bp, 1 kb, and 2kb.
